# Progress in Medicine

**Published:** 1897-01-23

**Authors:** 


### Progress in Medicine.

### DISEASES OF THE STOMACH.

**Gastrectasis.**—Curran Pope[1] divides cases of this disease into those caused mechanically, as for instance, by cicatrices; and those of functional origin, as in chlorotic and cardiac affections. He holds that the latter class can alone be entirely cured. The symptoms include distress and pain sometime after food, great flatulence, vomiting of food taken one or two days before, and many nervous and skin symptoms. There is increased prominence in the lower part of the gastric region, and sometimes splashing sounds and an enlarged area of dulness. The stomach tube brings up quantities of undigested food when used before breakfast or eight hours or more after a meal, and this usually contains much organic acid and fungi. As to treatment, he insists on a carefully chosen diet with little fluid at meal times, between which the stomach should have complete rest for several hours. Lavage should be carried out thoroughly once a day; massage, active exercise, and intraventricular faradisation are also of value. For any hyperchlorhydria or other conditions present the usual medicines may be given.

**Enteroptosis**, or Glenard's disease, the displacement of the stomach and other viscera, may result from tight-lacing, or follow pregnancy or the removal of a tumour. The altered position of the stomach is made out by percussion and auscultation, which may show at the same time the absence of dilatation. F. Gardner[2] says a provisional diagnosis may be made with the patient in the dorsal position from the depression which is seen above the umbilicus and a corresponding prominence below it. Many of these cases are regarded as instances of "pulsating aorta," from the stomach being thus pushed down. Abdominal supports, lying down while the stomach contains a load of food, and occasionally a week in bed will relieve the dragging pains which are often complained of. To prevent tight-lacing is generally beyond our power.

**Phlegmonous Gastritis.**—Another case of this rare disease is reported by Leith,[3] who has analysed fifty-two others. The walls are greatly thickened, the submucous coat being chiefly affected, light-coloured, and often semi-fluid. The serous covering is often unaffected, but peritonitis is not uncommon. Males are affected five times as often as females. As to bacteriology, we find that in Leith's own case it appeared to be a streptococcal disease. The symptoms are a sudden attack of abdominal pain, vomiting, pyrexia, tenderness over the stomach, restlessness and anxiety, while the termination has always been in death, so far as is known. Quinine has been recommended, and the streptococcal serum may be tried.

**Gastric Varix** is not very uncommon in cirrhosis of the liver; but E. Le. C. Lancaster reports an instance of death from rupture of varicose veins in the stomach in a healthy married woman, whose liver was quite normal. Several hæmorrhages without any pain occurred during a fortnight, ending in death.

**Mucous Polypi** in the stomach is another rare condition which was discovered by J. Barr Stevens'[5] post-mortem in the stomach of an epileptic. He had only twice complained of abdominal pain, but the fits were preceded by an aura commencing in the stomach, and there seemed to be at times distension of that organ. A dense mass of soft polypi grew from the lower part of the posterior wall, and some of these may have been pushed into the pylorus like a ball-valve during contractions. Dieulafoy[6] discusses a case of ulcer of the stomach where the symptoms till the final perforation were almost entirely absent, as in the case of varix, above mentioned. Even indigestion had not been noticed, and laparotomy was attempted too late to be of service. He remarks that the possibility of a latent ulcer of the stomach should always be considered in sudden attacks of peritonitis.

The effect of injuries such as strains and falls in the production of gastric ulcer or hæmatemesis is discussed at length by Ebstein,[7] who reports several cases. Three showed hæmatemesis and finally carcinoma after a strain, where he thinks a submucous hæmorrhagic infiltration may have led to digestion of the injured area; and ten presented ulcers after severe falls, possibly from vaso-motor changes. Local injuries, again, may be the starting point. Such a case resulted from swallowing a rusty nail. Schüle[8] has inquired what are the early signs of carcinoma. The absence of hydrochloric, the presence of lactic acid, and great motor inefficiency of the stomach are of value, especially when combined; but they may accompany a simple stenosis resulting from ulcer, and the secretory changes alone may be found in catarrh or enteroptosis. Boas and Hayem regard the secretory disorders as due to anatomical lesions, while Leube and his school believe they often depend on functional and nervous changes. Einhorn,[9] experimenting upon this point, found that the acid secretion does not correspond to the microscopical state of the mucosa, though proliferation of the glands is often met with in hyperchlorhydria and atrophy in achylia. Even these changes may be due to increase or decrease of the function, and ultimately to nervous causes. On the whole, his evidence is against an anatomical basis for these disorders, as the changes are only occasionally present. As an instance of the opposite view, we may refer to Hayem's[10] discussion of degenerative gastritis, which he divides into two types, fatty and inflammatory. The latter may present clear spaces among the epithelial cells, or there may be necrobiotic changes in them.

One little-known function of the stomach is that of excreting into its cavity various poisons circulating in the blood. Bongers[11] finds that a large number of drugs are thus excreted after rectal or subcutaneous injection, and when in large doses this may go on for days. Such drugs are morphia, caffeine, quinine, salicylates, and, according to Leineweber, strychnia and atropine. The process may commence in a quarter of an hour after injection of the poison. What is the ultimate destination of the substance, if not removed by vomiting or washing out the stomach, is not clear.

The Edinburgh Hospital reports recently contained an interesting paper on the symptoms produced by air swallowing and the sucking of air into the stomach. G. A. Sutherland[12] reports a case of the latter habit which led to great loss of weight and sleep. The trouble began with an attack of gastric catarrh, and consequent flatulence and discomfort. To relieve this the patient commenced the practice, as he thought, to expel the wind. When the chest is strongly expanded, if the glottis is kept closed and the neck muscles are fixed, a negative pressure is formed in the œsophagus and air is sucked in. This is readily returned with a loud noise, and the patient imagines that the flatus has been discharged from the stomach. Treatment of the stomach irritation should be accompanied with directions not to eructate by the mouth.

The action of saliva in digestion has generally been regarded as confined to changing starches in the mouth and, for a short time, in the stomach. Biernacki had, however, shown that it increases the acid and peptic powers of the stomach, and Friedenwald[13] has carried out of late elaborate experiments on the subject. He disproves Biernacki's strange idea that the saliva reduces the alkalinity of the food, but he shows abundantly that it markedly aids gastric digestion in healthy and in diseased conditions. This influence is not due to the amylotic action of the saliva, nor to the ptyalin, mastication, or the sulphocyanide of potash it contains, though the effect on the food is produced in its passage through the mouth and may be partly due to the carbon-dioxide swallowed. The details of the numerous experiments are difficult to summarise, but it appears that when saliva is swallowed with the test-meals, the free acid, the motor activity of the stomach, and generally the pepsin and rennet secretions are greatly increased, and proteid digestion is much more powerful. These results are certainly most remarkable, and seem to be well substantiated.

The subject is quite distinct from that of the salivary action on starches, and the so-called vegetable dyspepsia, where the absence of the action permits fermentation of farinaceous food in the stomach. W. A. Walker[14] pleads for the practice of adding diastase to the flour in bread-making, and the administration of taka diastase with meals when this form of dyspepsia occurs. The older remedy, papain, has been recently tested by Grote,[15] who finds it is undesirable in hyperacidity, and where ulcer or cancer is present, but it may be tried in cases of hypoacidity and deficient motor power.

The use of such remedies and of peptonised foods, however valuable, may, if too long continued in any stomach disorder, lead to ultimate enfeeblement of the digestive powers. H. A. Hare[16] points out the necessity of stopping the use of predigested foods as soon as possible, and refers to the experiments of Sir W. Roberts as showing the impaired powers of digestion which follow their long-continued use. Partially-digested food, except in extreme cases, is preferable to that completely changed, as it keeps in exercise any remaining natural powers. In connection with these dangers from over treatment it may be useful to note D. R. Paterson's[17] warning against the abuse of internal antiseptics. Some of them lessen the motor and acid functions, and may entirely destroy the enzymes both of the stomach and pancreas, thus seriously interfering with nutrition. In such a disease as typhoid this is a very serious matter, and may well be considered in discussing their advisability. This, too, of course, diminishes the action of natural antiseptics of the intestinal tract. The writer, however, finds in calomel an efficient agent which does not interfere with the digestive ferments. Other modes of obtaining some degree of intestinal asepsis are purgation and change of diet, and especially one of a carbohydrate type. Among other therapeutic suggestions, we must notice Sir J.

Sawyer's[18] praise of drachm doses of glycerine in the treatment of painful gastric digestion, such as are usually attributed to chronic catarrh of the stomach; Hershey's[19] reference to bromide of strontium, in twenty or thirty grain doses at meal times, in disorders of the stomach due to a nervous origin. He claims that there are few which it will not benefit at once, and without disagreeable after-effects. He also argues for the administration of very large doses of hydrochloric acid where it has been proved to be deficient. Pleiner's treatment of ulcer by very large doses of bismuth and lavage has been modified by Cramer,[20] who simply gives the bismuth to the patient to swallow before a meal, making him lie on the supposed site of the ulcer, so that the bismuth may gravitate and form a protective coat for it. The use of these large doses (150 to 300 grains) is not without danger, and Von Ziemssen speaks against them, and the use of the tube in administering them. Max Weiss[21] reviews the treatment of stomach disorders by electricity, and recommends internal treatment by galvanism for superacidity and by faradism for hypoacidity. Motor insufficiency often yields to internal faradism, which may be combined with massage. Many other methods are also discussed by him. In the treatment of enteroptosis Günzburg,[22] after considering massage, belts, electricity, diet, &c., relates that he has had good effects from the regular administration of small quantities of yeast, unless there is present any dilatation of the stomach. Robin[23] treats some forms of hyperchlorhydria by magnesia, three or four hours after a meal, or occasionally by milk or new egs at a similar time. Milk diet may be given for a week, to be followed by a regulated one.

In hypoacidity he rarely gives hydrochloric acid, but administers a carbohydrate diet, with soda, bitters, and ipecacuanha, and condiments. Hydrogen dioxide, or rather, hydrozone, is recommended by J. Aulde[24] in all forms of gastritis. He gives two to four ounces of a 1 in 33 solution of hydrozone half an hour before meals. The patient remains lying on his right side to favour the discharge of the disintegrated mucus through the pylorus. Then a liquid meal is given, and as improvement takes place solids may be carefully added. Glycozone is also given after meals to patients suffering from gastritis. In intestinal diseases injections of hydrozone are also recommended.

[1] Amer. Med. and Surg. Bulletin, May. [2] Med. Chronic., Oct. [3] Edinb. Hosp. Rep., 1896. [4] Lanc., Oct. 31. [5] Glasgow Med. J., June. [6] N. Y. Med. Jour., Aug. 15. [7] Int. Med. Magaz., Aug. [8] B. M. Jour., Oct. 31. [9] N. Y. Med. Rec., June 27. [10] Med. Week, Oct. 30. [11] Med. Chronic., Nov. [12] Lancet, Aug. 1. [13] Int. Med. Magaz., Aug. [14] Therap. Gaz., Sept. 15. [15] B. M. J., Aug. 22. [16] Therap. Gaz., Sept. 15. [17] Practit., Nov. [18] Lancet, July 4. [19] Therap. Gaz., Aug. 19. [20] N. Y. Med. Jour., Aug. 8. [21] B. M. J., Nov. 7. [22] B. M. J., Oct. 31. [23] Americ. J. Med. Sciences, Aug. [24] N. Y. Med. Jour., Aug. 15.

